# Replication of a local record keeping method for collecting road crash data in low resource settings: lessons from Bangladesh and Nepal

**DOI:** 10.1136/ip-2024-045279

**Published:** 2024-06-11

**Authors:** Martijn Thierry, Anish Khadka, Kazi Burhan Uddin, John Parkin, AKM Fazlur Rahman, Sunil Kumar Joshi, Julie A Mytton

**Affiliations:** 1 Safe Crossings, Huizen, Netherlands; 2 University of Auckland, Auckland, New Zealand; 3 Centre for Injury Prevention Research Bangladesh, Dhaka, Bangladesh; 4 University of the West of England—Frenchay Campus, Bristol, UK; 5 Kathmandu Medical College, Kathmandu, Nepal

**Keywords:** Motorcycle, Motor vehicle - Non traffic, Motor vehicle - Occupant, Pedestrian, Community Research, Low-Middle Income Country

## Abstract

**Background:**

Police road crash and injury data in low-income and middle-income countries are known to under-report crashes, fatalities and injuries, especially for vulnerable road users. Local record keepers, who are members of the public, can be engaged to provide an additional source of crash and injury data.

**Methods:**

This paper compares the application of a local record keeper method to capture road crash and injury data in Bangladesh and Nepal, assesses the quality of the data collected and evaluates the replicability and value of the methodology using a framework developed to evaluate the impact of being a local record keeper.

**Outcome:**

Application in research studies in both Bangladesh and Nepal found the local record keeper methodology provided high-quality and complete data compared with local police records. The methodology was flexible enough to adapt to project and context differences. The evaluation framework enabled the identification of the challenges and unexpected benefits realised in each study. This led to the development of an 11-step process for conducting road crash data collection using local record keepers, which is presented to facilitate replication in other settings.

**Conclusion:**

Data collected by local record keepers are a flexible and replicable method to understand the strengths and limitations of existing police data, adding to the evidence base and informing local and national decision-making. The method may create additional benefits for data collectors and communities, help design and assess road safety interventions and support advocacy for improved routine police data.

**WHAT IS ALREADY KNOWN ON THIS TOPIC**

- Under-reporting and bias are common limitations of police reported crash data in low-income and middle-income countries and are a major barrier to improving road safety.

**WHAT THIS STUDY ADDS**

- The local record keeper methodology is a replicable tool with the flexibility to be adapted to specific contexts to achieve high-quality data. The local record keeper methodology may yield additional benefits for local communities, such as increased awareness and commitment to road safety improvement.

**HOW THIS STUDY MIGHT AFFECT RESEARCH, PRACTICE OR POLICY**

- Researchers should consider using local record keeper methods for road safety research where routine sources of crash and injury data are considered unreliable. Outputs from such studies may evidence the need for, and enable advocacy for, improved quality of routine data collection and provide essential input for the design and assessment of road safety interventions.

## Background

 Police records are the main source of road crash and injury data in many low and middle-income countries (LMICs), but the data often have limitations, particularly under-reporting of crashes, fatalities and injuries, with specific under-representation of vulnerable road users (VRUs).^1 2^ Consequently, the burden of injuries and fatalities is not known accurately, and the proportion of VRU injuries and fatalities is likely to be higher than reported.^3^ The lack of complete and reliable road crash data is a problem because it hinders both research (eg, understanding the local epidemiology or the determination of the effectiveness of interventions) and policymaking (having evidence to inform decision-making and the ongoing monitoring of policy impacts).

Most road crash data used in research are either collected by researchers through primary studies or are routinely collected information that is accessed by researchers for secondary data analysis. Citizen science approaches, particularly in the arena of environmental research, have championed the use of members of the public as data collectors.^4 5^ In contrast, similar literature from health research tends to be limited to participatory research methods; for example, across research addressing communicable^6^ and non-communicable^7^ diseases. One area where this approach has been seldom used is road safety research.

A method to train members of the community to collect road crash and injury data was developed in 2017 and these ‘local record keepers’ (LRKs) were first engaged and reported in a road safety intervention study in Bangladesh.^8 9^ The methodology was then used in a separate study in Nepal.^10^ This paper aims to explore considerations concerning the use of the LRK methodology and provide advice on its replication as a research technique in other LMICs where the quality of routinely collected crash data is unclear or poor. Nosek and Errington^11^ state that ‘the purpose of replication is to advance theory by confronting existing understanding with new evidence’. Therefore, we evaluated the LRK methodology using a published theoretical framework.

## Methods

To compare the aims and the application of the LRK method in Bangladesh and Nepal and evaluate the data obtained, we conducted a documentary analysis^12^ of the study materials used in each setting, including study protocols, crash recording forms and analysis plans. We interpreted these findings based on our contextual understanding of these studies and our observations noted during visits to the study sites. Structured conversations with LRKs took place in both Bangladesh and Nepal as part of process evaluations in each study. We thematically analysed data from conversations with five LRKs in Bangladesh and six LRKs in Nepal using methods described by Braun and Clarke.^13^

We structured the evaluation using a framework developed by Roberts *et al*.^14^ Using qualitative methods, Roberts *et al* explored the experiences of data collectors working in communities exposed to poverty and violence. They described three themes: (1) *barriers*, the difficulties and challenges that data collectors encounter that may impact the data collection process, (2) *boundaries*, the data collector–participant relationship and the challenge of being both an impartial data collector while also a member of the community being evaluated and (3) *breakthroughs*, the unanticipated impacts for individuals and communities arising as a consequence of their involvement in the research process.

## Results

### Comparison of aims, settings and evaluation of the two studies

The aims of using the LRK methodology differed between the two studies. In Bangladesh, they were: (1) to derive relevant input for the design of a speed management intervention, (2) to evaluate intervention effectiveness and (3) to compare the LRK and police data. In Nepal, the study aimed to assess the quality and completeness of police crash reporting. In Bangladesh, two LRKs were based in each of three villages along a major highway and recorded crash events over a maximum of 1 km of road. In Nepal, the six LRKs were all shopkeepers with a business facing a busy highway where ribbon communities were common. Each shopkeeper recorded crashes occurring on approximately 500 m of road outside their shop.

A description of the study locations, including their selection, traffic mix and average speeds, together with the LRK crash data, are reported in Thierry *et al*^8^ and Khadka *et al.*^10^ Here, we present additional comparative analyses. The LRK methodology enabled nearly complete data in the crash records submitted by the LRKs; database fields were complete for 96.8% of crashes in Bangladesh and 99.5% in Nepal. Crash reporting rates and police attendance rates were calculated in both studies (table 1). When calculating reporting rates, the LRK data and police data each acted as the benchmark for the other.

**Table 1:** LRK and police reporting rates and police attendance rates, by country and by crash type

Study	Crash type	Reporting rate		Police attendance rate
		LRK	Police	
Bangladesh	Crashes involving any injuries	100%	2%	10%
	Fatal crashes	100%	18%	41%
Nepal	Crashes involving any injuries	89%	16%	49%
	Fatal crashes	63%	100%	100%
	Vehicle damage only crashes*	93%	12%	28%

* Vehicle damage only crashes were not recorded in Bangladesh.

LRKlocal record keeper

The reporting rates were estimated by matching the crashes recorded in the LRK data with the crashes recorded in the police data. Police attendance and reporting rates were higher in Nepal than Bangladesh. The rate of police attendance at crashes was higher than the rate of crashes reported by the police in both locations, which was an unexpected finding indicating that police records are not always completed when the officer returns to base. In both locations, the police are more likely to attend and report fatal crashes than those with injuries only. Nepali police are stationed at hospitals where they help file crash reports, so they are more likely than an LRK to be aware when a victim dies away from the crash scene. LRK reporting rates are higher in Bangladesh than Nepal, but low police reporting in Bangladesh means there is a lower probability of a crash appearing in the police data but not the LRK data.

In both studies, we collected data on fatalities and injuries by road user type (table 2). This type of analysis is important to identify road users who are more vulnerable to being injured or killed. In both Bangladesh and Nepal, people on motorcycles and three-wheelers were more vulnerable to injury and death. Pedestrians were vulnerable in both locations, but particularly in Bangladesh, which may reflect the faster average traffic speeds on the roads in the study sites in Bangladesh.

**Table 2:** Percentage of fatalities and injuries recorded by LRKs in Bangladesh and Nepal, by road user type

	Bangladesh		Nepal	
Road user type	% of fatalities(n=12)	% of injured people(n=274)	% of fatalities(n=5)	% of injured people(n=126)
Pedestrian	41.7	17.9	20.0	10.2
Rickshaw	0.0	5.8	0.0	0.0
Cyclist	0.0	7.3	0.0	0.8
Motorcycle	25.0	12.8	60.0	42.9
Motorised three-wheeler	25.0	35.8	0.0	11.9
Bus	0.0	7.7	20.0	28.6
Truck	8.3	9.1	0.0	3.2
Car/mini-bus/sumo/pick-up*	0.0	3.6	0.0	2.4
Total	**100.0**	**100.0**	**100.0**	**100.0**

Data from Bangladesh and Nepal should not be directly compared as differences between the two countries may reflect the differing vehicle fleets in use.

* In Bangladesh, mini-buses are mostly private 4four-wheeled vehicles hired by one family or organisation and have therefore been included with cars. In Nepal, mini-buses may also be 4four-wheeled vehicles but these are public transport vehicles and have therefore been included in the Bus category. Measurement period in Bangladesh: June 2013– - May 2014 (before implementation of the speed management programme). Measurement period in Nepal: 1 May 2019 – 30 April 2020.

LRKlocal record keeper

In Bangladesh, the LRK data showed that most crashes happened at the intersection between a highway and a side road and that buses were involved in half of fatal crashes. Three such intersections were subsequently chosen as a mid-point between speed humps that were installed as part of the intervention programme, and a design was selected that would reduce bus speeds.^8^ In Nepal, the LRK data illustrated the significant under-reporting of crashes, injuries and deaths in police records,^10^ which has been shared with the police and national government decision-makers and used to advocate for improvements in routine crash data.

### Evaluation of the methodology

The evaluation is presented using the framework developed by Roberts *et al*.^14^

#### Barriers

This theme captures the issues that may hinder the collection of good-quality data. Well-motivated data collectors are likely to be more diligent in their role and this will lead to more complete and accurately recorded data. Three of the LRKs in Nepal and three in Bangladesh reported that they felt that being an LRK was an opportunity to support their local community and they were very motivated to take up the role.

Pretesting of data collection forms with LRKs helped avoid incomplete data. In Bangladesh, LRKs shared concerns about particular questions, which led to these questions being amended or dropped. Of 14 data fields, only two had significant amounts of missing data: (1) the name of the hospital to which injured people were taken (22% missing, usually because this was not known by bystanders at the scene) and (2) the age and gender of the victim (9% missing, usually because the victims had left the scene before the LRK arrived). In Nepal, LRKs were concerned about the completion of data fields for multivehicle or complex crashes. These concerns were addressed with explanation from the LRK coordinator about how such events should be recorded.

In both studies, LRKs were not clinically trained and, therefore, it was not possible for them to accurately distinguish a minor injury from a serious injury. The recording of injury level was based on their judgement rather than the application of an injury severity scale. Caution should, therefore, be taken if comparing these data with studies using clinically assessed measures of severity. The Nepali crash recording form was simplified to include severity as ‘fatal’, ‘injured’ or ‘do not know’. LRKs could record a fatality only if it occurred at the scene since they were not able to follow-up victims after transfer to a hospital. This means that LRK data are not directly comparable with studies using the WHO’s definition of a fatality as being a person killed immediately or dying within 30 days, resulting from a road traffic collision.^15^

In both locations, LRKs were initially concerned they would miss crashes when they were away from their home or shop or not able to go to the crash site in person. However, in both studies, this risk was minimised by the connections that the LRK had within their community. In Bangladesh, the LRK sometimes asked other residents to record the crash data and pass data to them as soon as possible after the event. In Nepal, the LRK role was reported to be feasible for shopkeepers because there was usually someone else available (often a family member) to look after the shop, allowing the LRK to visit the crash scene.

All LRKs lived close to a busy highway and had witnessed multiple traffic crashes, injuries and deaths prior to recruitment to our study. Several LRKs had also actively provided assistance to crash victims. These experiences had normalised such events and instilled resilience towards traffic crashes and adverse outcomes. None of the LRKs reported, or was observed to experience, any emotional distress during the studies.

#### Boundaries

Roberts *et al*^14^ talk about the challenge of being an ‘impartial’ data collector. This was relevant in the Roberts' study which explored community violence, but was not a concern for LRKs because crashes were not seen as a sensitive topic and personal information such as the names of crash victims, were not recorded.

LRKs in both studies described how, during the initial stage of record keeping, members of the community did not understand the role the LRK had taken or the purpose of the data collection, resulting in occasional negative comments or challenges. In Nepal, the police questioned the authority and purpose of one LRK. In Bangladesh, the LRK coordinator supported awareness raising about the study and in Nepal, LRKs were provided with ID cards and study information sheets. Once the community and the police gained a better understanding of the local crash recording initiative, challenges stopped.

#### Breakthroughs

Roberts describes ‘Breakthroughs’ as unanticipated outcomes for data collectors and their communities. In both studies, recruiting and retaining LRKs were not difficult. People wanted to be an LRK to help their community, and they appreciated the financial compensation for their efforts. Retaining the LRKs for the duration of the studies was possible because of the motivation of the LRKs and the frequent and ongoing support from the LRK coordinator. In Nepal, all the LRKs were retained during the 1-year study, and in Bangladesh, LRKs were engaged for over 4 years as road safety interventions were implemented and evaluated.

LRKs in both studies reported beneficial impacts at a personal level. They described how they increased their knowledge of road crashes and why they occurred. In Nepal, three of the LRKs described how taking part led them to start considering how road traffic crashes could be prevented. All five of the LRKs in Bangladesh described how they were empowered to raise awareness in their local communities of how road users could keep themselves safe while crossing or using the road. The role motivated two Nepali LRKs to help crash victims when previously they may have only observed the crash. They also described how their role and knowledge were acknowledged and valued within their communities. These stories reveal how, by becoming a member of the research team, the LRKs had assumed an additional role within their communities. Two of the LRKs in Nepal and one in Bangladesh described how taking part in the study had given them additional status within the community and that members of the community would come to them to talk about road crashes.

In both studies, benefits for the local community were reported. In Bangladesh, community members turned to the LRKs to find out when the speed management programme would begin and what would happen. Communities were initially disappointed in the perceived low height of the speed humps that were built, but the LRKs were able to demonstrate their effectiveness by showing a reduction in crash frequency. In Nepal, three of the LRKs described feeling more confident to advocate for crash reduction in their community.

### Resulting proposed steps for replication

The WHO describe an eight-step process for establishing a police crash data system.^15^ Based on the evidence arising from our studies, we have adapted this guidance to develop an 11-step process for assessing the feasibility (steps 1–3) and subsequent implementation (steps 4–11) of the LRK methods in a new context (table 3).

**Table 3:** Proposed process to set up an LRK crash data system

Step number	Activity
1	Confirm objectives and desired output of the LRK methodology and review available routinely collected crash-related data
2	Recruit LRK coordinator or allocate this role to a member of the project team
3	Coordinator checks the feasibility of establishing LRKs: Can permission be obtained from relevant authorities? Is finding suitable LRKs realistic? Is the local community receptive to the idea of LRKs?
4	Design the LRK crash recording form and draft the LRK responsibilities
5	Design the data capture and follow-up process, including system requirements, project timeline and budget
6	Select and recruit the LRKs
7	Train the LRKs and pilot the crash recording form, data capture and follow-up processes
8	Collect the crash data with regular support and feedback from the LRK coordinator
9	Format data and apply quality control measures where necessary
10	Analyse crash data
11	Manage the end of the LRK data collection process

LRKlocal record keeper

Routinely available data should be reviewed (step 1) to determine whether the research question can be answered without further primary data collection. Key selection criteria for the LRK coordinator (step 2) include an understanding of the local context of road crashes in the study area (to align with local practices, infrastructure and socioeconomic factors), data collection, monitoring and evaluation experience (to ensure data quality), and the ability to engage with LRKs, local communities and authorities (to establish trust and rapport). Step 3 determines whether the methodology is feasible, based on whether permissions can be obtained from local authorities and the police, LRKs can be recruited and whether the local community will accept the record-keeping taking place. LRK selection criteria include an interest in road safety, living and working close to the road being monitored, a willingness and commitment to contribute to reducing road crashes, being resourceful and able to call on community support and having observational and recording skills. Community willingness may be tested through consultation meetings and discussions with elders, police, schoolteachers, and religious, political or community leaders.

The design of the crash recording form (step 4) is influenced by the study objectives and local circumstances (see examples in online supplementary materials). The data collection process is planned (step 5) before LRKs are recruited (step 6) and trained (step 7). Training should include piloting the data collection forms using dummy data, with modification of the forms based on LRK feedback where necessary. Data collection (step 8) with ongoing quality control (step 9) are facilitated by regular field visits and phone contact by the LRK coordinator to build strong relationships and collective problem-solving. To support LRKs, we recommend regular contact between LRKs and the LRK co-ordinator (a minimum visit frequency of once per month), encouraging LRKs to share their stories with friends, family and the local community and reminding the LRKs of the value of their contributions towards road safety. The process concludes with data analysis (step 10) and managing the end of study (step 11) which should include informing the LRKs of the value of their work and how the data will be used.

## Discussion and conclusions

We have evaluated an LRK methodology used in Bangladesh and Nepal for collecting road crash and injury data and described an 11-step process for researchers wanting to replicate the method in LMICs.

The availability of two sets of data (police and LRK) purporting to explain the same events (crashes, injuries and deaths) has high explanatory power for determining data quality. The need for valid road safety data underpins the Global Plan of Action for Road Safety 2021–2030^16^ which describes the responsibilities of governments to ensure capacity for the collection and analysis of data on crashes, deaths and injuries so that progress against the global target can be measured. Guidance on how to establish effective road safety data systems has been available for more than a decade.^15^

We note the percentages of VRUs involved in fatal crashes recorded by LRKs in these studies (92% of all fatalities in Bangladesh, and 80% in Nepal) are exceptionally high, and for Nepal higher than those reported in the latest Global Status Report on Road Safety at 60.3%.^17^ Deaths by road user category are not available for Bangladesh in the latest Global Status report for comparison. While our studies are only collecting estimates of road fatalities on small sections of roads and are only able to record deaths occurring at the scene of the crash, they illustrate the need for robust national-level data on deaths by road user group. We were surprised to find multiple instances where the LRKs reported that the police had attended a crash event, but we found no record of that crash in the police records. This under-reporting warrants further investigation, so that the issues faced by the police that prevent them from completing crash reports or records can be addressed.

The ability of LRKs to also record non-fatal injuries facilitates a local estimate of the total burden of road crashes and identifies which groups are most affected. Other studies have attempted to validate official police records using linked healthcare records,^18 19^ though this relies on the availability of robust health system records suitable for linkage and is, therefore, not feasible in many LMICs.

In a meta-synthesis of ways to improve data quality in road traffic injury registries, Sadeghi-Bazargani and colleagues emphasised the importance of ongoing support and communication.^20^ This aligns with our finding that the close working relationship between the LRKs and the LRK coordinator was crucial to achieving the high data completion rates. The relationship between community data collector and their community is equally important. Traditional road safety education interventions while having the ability to improve knowledge have been shown to have limited impact on reducing road traffic crashes.^21^ In our study, we identified the wider social value of using LRKs who shared their knowledge of road safety with their local community, thereby raising awareness and stimulating the potential for community-level advocacy for safer roads. The reported two-way sharing of knowledge between the LRK and their community and the potential benefit of the LRK model to foster context-relevant road safety education through a peer-based learning approach warrants further research.

Achieving a 50% global reduction in road traffic deaths and injuries by 2030 will require all countries to have effective data systems to monitor progress and evaluate the impact of policies and interventions.^17^ Most LMICs already record road traffic deaths and injuries using police reports, but the data are known to underestimate crashes and injuries. Assuming the criteria above can be met, we conclude that the LRK methodology is a flexible and replicable method for LMICs to understand the strengths and limitations of existing police data and, where necessary, inform action to strengthen official data. For example, the LRK method could aid the identification of data gaps, and replication of the LRK method across a representative set of roads could enable modelling to derive a more accurate estimate of crashes and injuries. Such outputs would add to the evidence base and inform local and national decision-making.

## supplementary material

10.1136/ip-2024-045279 online supplemental file 1

## Data Availability

All data relevant to the study are included in the article or uploaded as supplementary information.
