# Supplementary material for: Replication of a local record keeping method for collecting road crash data in low resource settings: lessons from Bangladesh and Nepal
Source: Inj Prev. 2024 Jun 11;30(5):e045279. doi: 10.1136/ip-2024-045279 (PMC11503161; doi:10.1136/ip-2024-045279)
Supplement: online supplemental file 1 [file ip-30-5-s001.pdf]

Replication of a local record keeping method for collecting road crash data in low resource settings: lessons from Bangladesh and Nepal

SUPPLEMENTARY MATERIALS

Bangladesh road traffic crash recording form

1 ID No:
2 Date of crash:
3 Time of crash:
4 Village: 1. Nama Para 2. Nil Kuthi 3. Kunder Para
5 Place of crash 1. Intersection 2. Very close to Intersection 3. Further away from intersection
6 Type of crash: [draw line to connect road users involved in crash]

Bus	Bus
Minibus	Minibus
Micro bus/car	Micro bus/car
Truck/Lorry	Truck/Lorry
CNG	CNG
Motor Bike	Motor bike
Tempoo/Nosimon	Tempoo/Nosimon
Rickshaw	Rickshaw
Pedestrian	Pedestrian
Others (Specify) ________________	Others (Specify) _________________

7 Number of persons injured?
8 Number of person(s) died?
9 Police visited the spot after accident? 1. Yes 2. No 3. Don't Know
10-14 Age, sex and other information (for each crash victim (each fatality and each injured person)

Sl No.	Age	Sex	Type of injury	Admitted to the hospital?	Name of hospital
01					
02					
03					
04					
05					
Etc.					

Name of record keeper: _____________________________________

Nepal road traffic crash recording form

Data collector ID:

Crash number:

1 Date of crash: YYYY/MM/DD
2 Time of crash: hh:mm and morning / afternoon / evening /night
3 Weather at time of crash: rain / fog or mist / not raining or foggy
4 Road condition at time of crash: road wet / road dry
5 Where did the crash happen? (description)
6 Number of vehicles involved:
7 Number of driver casualties: (if no driver casualties, put 00)
8 Number of passenger casualties: (if no passenger casualties, put 00)
9 Number of pedestrian casualties: (if no pedestrian casualties, put 00)
10 Vehicle damage only: Yes / no (if there were no casualties, and there was only vehicle damage, tick yes)
11 Crash type (please circle the most appropriate option):
    1 collision between moving vehicles;
    2 vehicle turned over on the road;
    3 vehicle fell off the road;
    4 hit pedestrian;
    5 hit cyclist;
    6 hit animal;
    7 hit parked vehicle;
    8 hit object on the road;
    9 unknown;
    10 other (please state)
12 Vehicle types involved in the crash (please circle as appropriate):
    Vehicle 1: 1 bicycle, 2 rickshaw, 3 motorcycle, 4 scooter, 5 car/jeep/sumo, 6 pick-up, 7 mini-bus, 8 tractor, 9 truck, 10 tanker, 11 tipper, 12 auto-rickshaw/tempo/magic, 13 unknown, 14 other (please state)
    Vehicle 2: (as above)
13 Did the police visit the crash site: yes / no / do not know
14 Short description of what happened in the crash: (description)
15 Information on injured people:

	**Age**	**Sex**	**Injuries at the scene**	**Taken to hospital**
	1 pre-school<br>2 school age<br>3 adult<br>4 do not know	1 male<br>2 female<br>3 do not know	1 fatal (died at the scene)<br>2 non-fatal injuries<br>3 do not know	1 yes<br>2 no<br>3 do not know
1				
2				
3				
4				
5				
6				

(Use the back side of the form to add more injured people)

Signature of record keeper: ____________________________________
